# Cultivation of Bacteria From *Aplysina aerophoba*: Effects of Oxygen and Nutrient Gradients

**DOI:** 10.3389/fmicb.2020.00175

**Published:** 2020-02-19

**Authors:** Johanna Gutleben, Catarina Loureiro, Laura Adriana Ramírez Romero, Sudarshan Shetty, René H. Wijffels, Hauke Smidt, Detmer Sipkema

**Affiliations:** ^1^Laboratory of Microbiology, Wageningen University & Research, Wageningen, Netherlands; ^2^Bioprocess Engineering, AlgaePARC, Wageningen University, Wageningen, Netherlands

**Keywords:** microbial cultivation, marine sponge, *Aplysina aerophoba*, viability PCR, antibiotic resistance, environmental microbes

## Abstract

Sponge-associated bacteria possess biotechnologically interesting properties but as yet have largely evaded cultivation. Thus, “omics”-based information on the ecology and functional potential of sponge symbionts is awaiting its integration into the design of innovative cultivation approaches. To cultivate bacteria derived from the marine sponge *Aplysina aerophoba*, nine novel media formulations were created based on the predicted genomic potential of the prevalent sponge symbiont lineage Poribacteria. In addition, to maintain potential microbial metabolic interactions *in vitro*, a Liquid-Solid cultivation approach and a Winogradsky-column approach were applied. The vast majority of microorganisms in the inoculum appeared viable after cryopreservation of sponge specimen as determined by selective propidium monoazide DNA modification of membrane-compromised cells, however, only 2% of the initial prokaryotic diversity could be recovered through cultivation. In total, 256 OTUs encompassing seven prokaryotic phyla were cultivated. The diversity of the cultivated community was influenced by the addition of the antibiotic aeroplysinin-1 as well as by medium dilution, rather than carbon source. Furthermore, the Winogradsky-column approach reproducibly enriched distinct communities at different column depths, amongst which were numerous Clostridia and OTUs that could not be assigned to a known phylum. While some bacterial taxa such as *Pseudovibrio* and *Ruegeria* were recovered from nearly all applied cultivation conditions, others such as Bacteroidetes were specific to certain medium types. Predominant sponge-associated prokaryotic taxa remained uncultured, nonetheless, alternative cultivation approaches applied here enriched for previously uncultivated microbes.

## Introduction

Marine sponges represent the oldest, living lineage of the animal kingdom, with a longstanding association to microorganisms (Hooper and Van Soest, 2012; McFall-Ngai et al., 2013). To date, 41 prokaryotic phyla have been found in association with sponges (Thomas et al., 2016) and accordingly, this vast genetic potential is hypothesized to be accountable for numerous interactions between sponge symbionts and their hosts (Webster and Thomas, 2016; Moitinho-Silva et al., 2017; Chaib De Mares et al., 2018). In recent years, omics-based methods (Horn et al., 2016; Slaby et al., 2017) as well as physiological *in situ* studies have shed some light on microbial processes in sponges (Bayer et al., 2008; Becerro et al., 2012). Microbes filtered from the seawater comprise the primary food source for most sponges, whilst specific microbes evade digestion by the sponge cells and get established in the mesohyl where they grow on metabolic waste products or host-derived carbohydrates (Vogel, 2006; Taylor et al., 2007; Simpson, 2011; Kamke et al., 2013; Bayer et al., 2018). Additionally, carbon fixation by photosynthesis (Burgsdorf et al., 2015), nitrification (Bayer et al., 2008; Hoffmann et al., 2009), sulfur cycling (Keren et al., 2015), phosphorus cycling (Zhang et al., 2015), vitamin synthesis by microorganisms, and prokaryotic production of secondary metabolites for host defense (Kennedy et al., 2007; Hochmuth and Piel, 2009; Freeman et al., 2012; Indraningrat et al., 2016) all occur in the sponge holobiont (Webster and Thomas, 2016). However, detailed disentanglement of prokaryotic functionalities is majorly hindered by the lack of cultured representatives of sponge-associated bacteria and archaea. Despite numerous approaches, none of the predominant sponge associated phylotypes belonging to the Acidobacteria, Chloroflexi, Cyanobacteria, Nitrospirae, Poribacteria or Thaumarchaeota could be cultivated *in vitro* (Sipkema et al., 2011; Lavy et al., 2014; Steinert et al., 2014; Keren et al., 2015; Versluis et al., 2017). One of the reasons might include the inability to recreate sponge-mesohyl conditions adequately, since sponges and their microbiomes evolved complex networks of cross-feeding and other interactions (Pande and Kost, 2017). By using conventional cultivation approaches, such interaction networks are mostly disrupted during the early stages of isolation. Recent advances in multi-omics techniques, however, allow insights into the genomic and metabolic potential of uncultured microorganisms. Integrating such information can reduce the search-space inherent to cultivation experiments, which might prove highly valuable for facilitating the cultivation of novel microbial lineages (Gutleben et al., 2018).

The Mediterranean sponge species *A. aerophoba* poses an interesting model for investigating the cultivability of sponge associated microorganisms due to its association with a highly diverse microbial consortium (Hentschel et al., 2002; Schmitt et al., 2012; Slaby et al., 2017). Furthermore, this sponge exhibits a rich biochemical arsenal comprising of high amounts of brominated alkaloids (Turon et al., 2000), which correlate with the abundance of certain prokaryotic taxa (Sacristán-Soriano et al., 2011, 2016). Since FADH_2_-dependent halogenase gene fragments of microbial origin have been detected in *A. aerophoba*, microorganisms might be the actual producers of such brominated bioactives (Bayer et al., 2013). One example is the antibiotic aeroplysinin-1 (AP), protecting damaged sponge tissue from bacterial infections (Ebel et al., 1997; Thoms et al., 2004; Niemann et al., 2015). Despite various cultivation approaches utilizing antibiotics (Pimentel-Elardo et al., 2003; Sipkema et al., 2011; Versluis et al., 2017), the inclusion of sponge-derived antimicrobials as a selection criterion has remained scarce.

A predominant fraction of the *A. aerophoba* microbiota is constituted by members of the bacterial candidate phylum Poribacteria. This ubiquitous and widely distributed sponge-associated phylum represents a phylogenetically distant member of the Planctomycetes-Verrucomicrobia-Clamydiae superphylum (Fieseler et al., 2006; Wagner and Horn, 2006). While poribacteria have remained recalcitrant to cultivation despite multiple approaches (Pimentel-Elardo et al., 2003; Hardoim et al., 2014; Lavy et al., 2014), recent cultivation-independent multi-omics studies illuminated on the lifestyle of this candidate phylum (Fieseler et al., 2004, 2006; Kamke et al., 2013, 2014; Jahn et al., 2016) postulating a heterotrophic, aerobic metabolism with the genetic potential to degrade a wide range of carbohydrates and glycoproteins.

Keeping the above in mind, this study aims to address the discrepancy between the cultivable and total community of *A. aerophoba* and investigates several issues potentially contributing to the current uncultivability of sponge-associated bacteria. Firstly, we investigate whether sample processing and cryopreservation impacts the viability of sponge-associated bacteria. Secondly, we explore the use of -omics data in defining nutrients and cultivation conditions for Poribacteria and the addition of a sponge derived antibiotic (aeroplysinin-1). Lastly, we describe the first attempts to enrich a complex microbial community maintaining at least some metabolic interactions of the sponge microbiome in a stratified cultivation system based on the Winogradsky-column approach (Madigan et al., 2014; Rundell et al., 2014) and within Liquid-Solid cultures.

Subsequently, 16S rRNA gene amplicon sequencing was used to investigate the diversity and composition of (i) the total prokaryotic community of *A. aerophoba*, (ii) its viable fraction after cryopreservation, and (iii) its cultivable fraction. With these approaches we aim to contribute to the understanding of the ecological and biotechnological properties of *A. aerophoba* and its associated microbiota.

## Materials and Methods

### Sample Collection and Sponge Tissue Processing

Three *A. aerophoba* individuals were sampled in June 2014 by SCUBA diving in Cala Montgó, Spain (42.1140N, 3.167E) between 7.8 and 12.7 m depth. The individual sponges all possessed several oscula and grew between 3 and 15 m apart from each other on sun-exposed rock surfaces. Approximately 5 cm^3^ of each individual were cut with a diving knife and transferred to 50 mL centrifuge tubes under water. Samples were kept on ice and transported to the laboratory within few hours. Tissue separation and cryopreservation of all individuals was performed as previously described (Sipkema et al., 2011), using a final concentration of 25% glycerol in sterile artificial sea water [ASW, 33 g/L (Reef Crystals, Blacksburg, VA, United States)] as cryoprotectant. Cryopreserved samples were transported to Netherlands and stored at −80°C for several months. Four seawater samples (2 L each) were collected at the sampling site in proximity to the sponges and filtered over 0.2 μm pore size polycarbonate filters. Filters were stored at −20°C until DNA extraction was performed.

### Viability of Microorganisms After Cryopreservation

**FIGURE 1:**
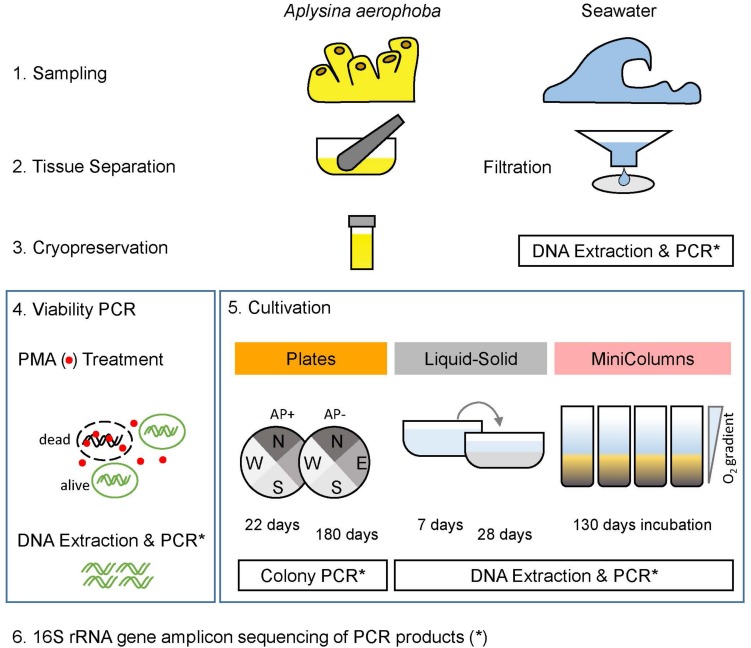
Experimental layout. Sponge samples (*A. aerophoba*) were cryopreserved and subsequently subjected to a viability test and three cultivation methods. For the (a) Plates cultivation experiment, colonies were picked after 22 days and 180 days of incubation. In (b) Liquid-Solid cultivation, cryopreserved material was incubated in liquid medium for 7 days, then transferred to Liquid-Solid medium for 28 days. In (c) Winogradsky columns (MiniColumns), the established oxygen gradient was assessed and samples were taken after 130 days of incubation. Prokaryotic diversity of all samples was determined by sequencing 16S rRNA gene amplicons.

A cryopreserved cell suspension of *A. aerophoba* (Aa18) was thawed and divided into four 150 μL aliquots. Two aliquots served as total prokaryotic community controls (Cryostock samples) and were stored at 4°C for a few hours until DNA extraction as described below. To assess the viable prokaryotic community after cryopreservation (Figure 1), two aliquots were treated with a propidium monoazide dye (PMAxx, Biotium Hayward, CA, United States) following manufacturer’s instructions (here referred to as PMA samples). PMA permanently modifies DNA of membrane-impaired, dead cells and thus only DNA from viable cells with intact membranes is amenable to PCR amplification and sequencing (Nocker et al., 2007; Emerson et al., 2017). After photo-activation of the dye (using the PMA-Lite LED Photolysis Device), cells were pelleted for subsequent DNA extraction.

### Cultivation Setup

#### (a) Plates

Based on single cell genomics, Kamke et al. (2013) postulated that Poribacteria can use a wide range of carbohydrates as energy source for their central metabolism. Poribacteria seem well adapted to degrade sponge mesohyl- and seawater-derived carbohydrates including compounds such as uronic acids, glucose, N-acetylgalactosamine, xylose and galactosides such as lactose and melibiose. The predicted carbohydrate degradation potential of Poribacteria (Kamke et al., 2013) served as a basis for the design of nine defined media formulations. The media differed only in carbon source, comprising of a variety of different polysaccharides and monomeric sugars. For each of the media, three carbon concentrations were applied: 1x (4 g/L), 10x diluted (0.4 g/L), and 50x diluted (0.08 g/L). Additionally, all media contained the same nitrogen, phosphorous and sulfur sources as well as micronutrients and trace elements (Table 1), which were defined in an attempt to meet metabolic needs of Poribacteria. All components were dissolved in artificial seawater (ASW). For medium dilutions (10x and 50x) all medium components were diluted except the micronutrients and trace metals, which were kept constant.

**TABLE 1:** Composition of nine different media used for the Plates experiment.

	Carbon source			Other nutrients	
		
Medium	Ingredients	[g/L]	Nutrient	Ingredients	[mg/L]
Glc	N-acetylgalactosamine	2	Nitrogen	NaNO_2_	250
	N-acetylglucosamine	2		Urea	250
Fru	Fucose	1	Phosphorous	Aminoethyl-phosphonate (2-AEPn)	250
	Fructose	1	Sulfur	Cysteine	40
	Glucose	1		Histidine	40
	Sucrose	1		Methionine	40
Gal	Lactose	1		NaSO_4_	80
	Galactose	1	Micronutrients	BME vitamin solution	1 mL
	D-galactonate	1		B2	2
	Melibiose	1		B9	2
Aa	All amino acids	2		B12	5
	N-acetylglucosamine	2		FAD	1
Col	Collagen coated	3		NAD	2
	Galactose	1	Trace metals	Customized trace metal solution	1 mL
Muc	Glycoprotein: Mucin from porcine stomach	4	Customized trace metal solution	for 1L	[mg/L]
Myo	Myo-inositol	4		FeCl_2_^∗^ 4 H_2_O	1500
Rha	L-rhamnose	1		7.7 M HCl	10 mL
	L-rhamonate	1		CoCl_2_^∗^ 6 H_2_O	190
	D-xylose	1		MnCl_2_^∗^ 4 H_2_O	100
All	N-acetylgalactosamine, N-acetylglucosamine,	0.30 each		ZnCl_2_	70
	Fructose, Sucrose, Glucose, Lactose,			H_3_BO_3_	6
	Melibiose, Galactonate, Galacturonate,			Na_2_MoO_4_	36
	Glucuronate, Mucin, Myo-Inositol,			NiCl_2_^∗^ 6 H_2_O	24
	Rhamnose, Xylose			CuCl_2_^∗^ 2 H_2_O	2
				MoO_2_	10
				KMnO_4_	190
				NaI	5
				KBr	5

*Concentrations are given for undiluted (1x) media. Carbon, nitrogen, sulfur and phosphorous sources were diluted for 10x and 50x diluted media, while other nutrients were kept constant in all media.*

Media were solidified using 0.75% (w/v) gellan gum (GELRITE, Carl Roth, Karlsruhe, DE), since agar has been shown before to impair the cultivability of environmental bacteria (Narihiro and Kamagata, 2013; Tanaka et al., 2014; Rygaard et al., 2017). Before autoclaving the pH was set to between 8 and 8.5, to achieve a final pH of approximately 8. After autoclaving, cycloheximide (250 mg/L, anti-fungal), carbon source, micronutrient and trace metal solutions, all of which were filter-sterilized, were added to the media and quickly poured in 90 mm diameter petri dishes. The collagen media plates were coated with 0.1% (1 mg/mL) solution of calf skin collagen (Sigma-Aldrich, St. Louis, MO, United States) after cooling according to the manufacturer’s instructions.

Plates were divided in quadrants, and kanamycin disks (30 μg Thermo Fisher Scientific, Waltham, MA, United States, Oxoid Kanamycin Antimicrobial Susceptibility Disks) were placed in the approximate center of each of the quadrants, to prevent the growth of *Pseudovibrio* spp. in limited areas of the plates (Versluis et al., 2017). For each medium and its three dilutions, three replicate plate sets were used for incubation with (AP_positive) and without (AP_negative) aeroplysinin-1, and a negative control.

A cryopreserved *A. aerophoba* (Aa16) cell suspension was thawed, serially diluted in sterile ASW from 10^–1^ to 10^–4^, and 50 μL of each dilution was inoculated on one of the quadrants of the plates. For incubation with a sponge-derived antibiotic (AP_positive), the cryopreserved sponge cell suspension (Aa17) was incubated with 5 mg/L of aeroplysinin-1 (Abcam, Bristol, United Kingdom, dissolved in DMSO), for 15 min at room temperature prior to inoculation. One plate of each medium type served as negative control and was inoculated with sterile ASW. The remaining sponge cell suspension (Aa16 and Aa17) was stored at −20°C for total community DNA extraction. Plates were incubated at 20°C in the dark and colonies were picked after 20–25 as well as after 180 days. None of the negative control plates showed growth after 25 days. No colonies could be observed on the collagen-coated plates Col_10x and Col_50x and they were discarded. A maximum of 48 individual colonies were picked per plate, starting with the highest inoculum dilution quadrant, thus preferentially picking colonies derived from highly abundant community members, and picking at least one representative of every discernible colony morphology. For each colony, media type and dilution, inoculum dilution, incubation time, morphology and distance from kanamycin patch was recorded (available upon request). Individual colonies were picked using 200 μL pipet tips and transferred to both a sterile 1 mL 50% glycerol solution and to 100 μL nuclease free water in 96 well plates. The glycerol stocks were cryopreserved at −80°C in cryovials, and nuclease free water was stored at −20°C in 96 well microtiter plates as template for 16S rRNA gene amplification by colony PCR.

#### (b) Liquid-Solid Media Cultivation

In order to investigate whether a liquid-solid interface within the cultivation medium would result in a different enriched community, 100 μL of cryopreserved *A. aerophoba* cell suspension (Aa18) was inoculated into liquid, 50x diluted “All amino acids medium” (Aa_50x medium) in duplicate. After 7 days of incubation, the biofilm which was attached to the bottom of the culture dishes was scratched-off the bottom, and the entire culture was harvested, 1 mL of the liquid-preculture was cryopreserved by adding 1 mL of 50% glycerol in ASW and 1 mL was stored at −20°C for subsequent DNA extraction. Another 100 μL were transferred to cell culture dishes containing solidified Aa_50x medium overlaid by 5 mL of liquid Aa_50x medium. This Liquid-Solid culture was incubated for 4 weeks. Subsequently, the entire cultivated microbial communities were collected by carefully scraping the established biofilm off the submerged gelrite surface, and 1 mL of total Liquid-Solid culture was cryopreserved as described above, while another 1 mL was stored at −20°C for DNA extraction.

#### (c) Winogradsky Columns

Winogradsky columns, hereafter referred to as MiniColumns, were prepared by filling glass culture tubes (25 mL) with 15 g of silicate sand (Sibelco, Antwerp, Belgium), and 75 mg of crystalline cellulose (5 mg/L) was mixed in as carbon source. For the water phase of the column 50x diluted marine broth (0.75 g/L, Thermo Fisher Scientific, Waltham, MA, United States), 0.5 g/L urea and 0.01 g/L NaSO_4_ were dissolved in ASW. The pH was adjusted to 7.5, then 15 mL of this medium (referred to as MiniColumn Medium) was added on top of the silicate sand and the MiniColumns were autoclaved. After autoclaving, 1 mL/L of phosphate solution (5 mg/L NaH_2_PO_4_) and 1 mL/L BME vitamins (Sigma-Aldrich, St. Louis, MO, United States) were added. Four replicate MiniColumns were inoculated with 300 μL of cryopreserved *A. aerophoba* sponge-cell suspension (Aa23). One MiniColumn was inoculated with sterile ASW as a negative control. The MiniColumns were closed with metal-caps and aluminum foil in order to allow for oxygen diffusion and incubated at room temperature (20°C) next to a north side window under natural daylight conditions. They were not moved any more in order not to disturb the gradient formation and microbial community development. After 130 days of incubation, an oxygen microsensor (PreSens, Regensburg, DE) was inserted into the columns, and oxygen concentrations were measured continuously from the water surface until the sediment fraction. Immediately afterward, the MiniColumns were divided into five samples per column representing the surface (WS), the top (WT) and the bottom (WB) of the water phase as well as the top (ST) and the bottom (SB) of the sediment fraction. The samples were cryopreserved as explained before, and 1 mL of water phase or 1 g of sediment was stored at −20°C for subsequent DNA extraction.

### DNA Extraction

Total community DNA was extracted from i. cryopreserved sponge and bacteria cell suspensions, ii. filtered environmental seawater samples, iii. the four aliquots of the viability tests, iv. samples from 7 days and 4 weeks of Liquid-Solid cultivation, and v. the MiniColumn samples (Figure 1). Picked colonies were directly used for PCR without prior DNA extraction. Seawater filters were cut into small fragments and suspended in 400 μL of STAR buffer (Roche Diagnostics Corporation, Indianapolis, IN, United States). Sediment samples of the MiniColumns were mixed with 1 mL of STAR buffer and vortexed thoroughly. After letting the sediment settle, the supernatant was transferred to bead-beating tubes. For all other sample types, cells were pelleted at 10 000 *g* for 10 min, resuspended in 400 μL of STAR buffer and transferred to 2 mL sterile bead-beating tubes filled with 0.1 g of 0.11 mm Zirconia beads (BioSpec Products, Bartlesville, OK, United States). Cell lysis was achieved by bead-beating using a Precellys 24 tissue homogenizer (Bertin Instruments, Montigny-le-Bretonneux, FR) for two times 30 s at a speed setting of 5.5 m/s. Total community DNA was extracted using a Maxwell 16 Instrument in combination with the Maxwell 16 Tissue LEV Total RNA Purification Kit customized for microbial DNA extraction (Promega, Madison, WI, United States) following manufacturer’s instructions. The DNA was eluted in 30 μL DNAse free water and quantified using Nanodrop 2000c Spectrophotometer (Thermo Fisher Scientific, Waltham, MA, United States) and gel electrophoresis.

### 16S rRNA Gene Amplicon Sequencing

Amplicons of the V4 region of the 16S rRNA gene were generated from extracted DNA, using a two-step PCR protocol. In the first step, PCR amplicons were generated using the primers 515f-806rB (Walters et al., 2015) with an attached linker sequence (UniTag) (van Lingen et al., 2017). PCR was performed in triplicate and the reaction mix contained 5 μL 5X Phusion HF buffer, 0.5 μL dNTPs (10 mM), 1 μL UniTag1-515f primer (5′-GAGCCGTAGCCAGTCTGC-GTGYCAGCMGCCGCGGTAA-3′) (10 μM), 1 μL UniTag2-806rB primer (5′-GCCGTGACCGTGACATCG-GGACTACNVGGGTWTCTAAT-3′) (10 μM), 0.25 μL Phusion Hot Start Polymerase (Thermo Fisher Scientific, Waltham, MA, United States), and 1–10 μL of extracted DNA (20 ng/μL). Nuclease-free water (Promega) was added to yield a total reaction volume of 25 μL. The PCR program comprised of initial denaturation at 98°C for 30 sec (10 min for colony PCR), followed by 25 cycles of denaturation at 98°C for 45 sec, annealing at 50°C for 30 sec, elongation at 72°C for 10 sec, and a final extension step at 72°C for 7 min. PCR products were visualized on agarose gel. In the second step PCR a sample-specific barcode was added as described below.

The colonies picked from the Plates experiment were also identified using 16S rRNA gene amplicon sequencing. First step PCR amplicons were generated directly by colony PCR, without prior DNA extraction. Each colony was PCR amplified following the protocol described above, in a single reaction using 1 μL of the nuclease free water template. All colony PCR reactions with a negative result were repeated. To reduce the number of samples for 16S rRNA gene profiling, on average 40 colony PCR products were pooled at approximately equimolar amounts (based on gel band intensity) into one sample for the second step PCR. For the AP_negative set of plates, one PCR product pool corresponded to the amplified colonies from one medium plate (Glc_1x – Rha_50x, Table 2). Due to the low yield of colonies from AP_positive plates, insufficient numbers of positive PCR products were available per pool, thus colonies from more than one plate were pooled to keep the number of PCR products consistent within one sample for 16S rRNA gene amplicon sequencing. These samples were labeled P13II to P19II (Table 2) and resulted in mixed carbon source and medium dilutions. All PCR products that resulted from colonies picked after 180 days of incubation were pooled into four samples for amplicon sequencing and labeled 2ndP1I to 2ndP2II (Table 2).

**TABLE 2:** Overview of samples derived from three cultivation experiments (cultivation samples) and associated meta data.

a) Plates	SampleID	Medium	Medium dilution	Aeroply snin (AP)	Incubation time [d]	PD	b) Liquid- solid	SampleID	Medium	Medium dilution	Type	Incubation time [d]	PD
	Glc_1x	Glc	1x	—	20	2.40		A7i	Aa	50x	Liquid-Culture	7	1.73
	Fru_1x	Fru	1x	–	20	2.48		A7ii	Aa	50x	Liquid-Culture	7	2.51
	Gal_1x	Gal	1x	–	20	2.29		Aw4i	Aa	50x	Liquid-Solid	28	1.79
	Aa_1x	Aa	1x	–	20	2.43		Aw4ii	Aa	50x	Liquid-Solid	28	2.32
	Col_1x	Col	1x	–	20	2.26							
	Muc_1x	Muc	1x	–	20	2.38	**c) MiniColumns**	**SampleID**	**Location**		**O2 conc. [%]**	**Inc., time [d]**	**PD**
	Myo_1x	Myo	1x	–	20	2.27		1WS	Water_Surface		65.9	130	3.08
	Rha_1x	Rha	1x	–	20	2.39		2WS	Water_Surface		41.2	130	3.08
	All_1x	All	1x	–	20	2.39		3WS	Water_Surface		36.8	130	2.85
	Glc_10x	Glc	10x	–	20	2.30		4WS	Water_Surface		6.8	130	1.99
	Fru_10x	Fru	10x	–	20	2.63		1WT	Water_Top		25.4	130	2.84
	Gal_10x	Gal	10x	–	20	2.99		2WT	Water_Top		26	130	3.00
	Aa_10x	Aa	10x	–	20	2.30		3WT	Water_Top		30.6	130	2.94
	Muc_10x	Muc	10x	–	20	2.70		4WT	Water_Top		1.8	130	2.76
	Myo_10x	Myo	10x	–	20	3.04		1WB	Water_Bottom		0.1	130	3.57
	All_10x	All	10x	–	20	1.95		2WB	Water_Bottom		15.3	130	3.00
	Glc_50x	Glc	50x	–	20	2.94		3WB	Water_Bottom		30.6	130	2.96
	Fru_50x	Fru	50x	–	20	2.30		4WB	Water_Bottom		1.8	130	3.06
	Gal_50x	Gal	50x	–	20	3.27		1ST	Sediment_Top		0.1	130	2.55
	Aa_50x	Aa	50x	–	20	3.21		2ST	Sediment_Top		6.4	130	2.48
	Muc_50x	Muc	50x	–	20	3.26		3ST	Sediment_Top		19.8	130	2.22
	Myo_50x	Myo	50x	–	20	2.94		4ST	Sediment_Top		0.4	130	2.20
	Rha_50x	Rha	50x	–	20	3.03		1SB	Sediment_Bottom		0.1	130	3.09
	P13II	Mix	mix	Mix	20	1.73		2SB	Sediment_Bottom		3.2	130	2.62
	P14I	Mix	mix	+	24	1.54		3SB	Sediment_Bottom		0.3	130	2.46
	P14II	Mix	mix	+	24	2.61		4SB	Sediment_Bottom		0.2	130	2.13
	P15I	Mix	mix	+	24	2.37							
	P15II	Mix	mix	+	24	2.34							
	P16I	Mix	mix	+	24	2.29							
	P16II	Mix	mix	+	24	2.35							
	P17I	Mix	mix	+	24	2.55							
	P17II	Mix	mix	+	24	2.68							
	P18I	Mix	mix	+	24	2.24							
	P19I	Mix	mix	+	24	2.74							
	P19II	Mix	mix	+	24	2.69							
	2ndP1I	Mix	mix	–	180	4.11							
	2ndP1II	Mix	mix	–	180	3.94							
	2ndP2I	Mix	mix	Mix	180	4.30							
	2ndP2II	Mix	mix	–	180	3.84							

*For (a) the Plates experiment, a cultivation sample corresponds to a pool of single colony PCR products, which, for the AP_negative set, corresponds to the colonies derived from one plate with a specific carbon source and medium dilution. Due to the low yield of colonies from the AP_positive set, positive PCR products from more than one plate were pooled (referred to as “mix”). For (b) the Liquid-Solid cultivation, a cultivation sample corresponds to the prokaryotic community that was enriched after 7 or 28 days of incubation. For (c) the MiniColumns, a cultivation sample corresponds to the community enriched in a specific fraction in one of the four replicate columns. PD, Faith’s Phylogenetic Diversity.*

Second step PCR reactions were done in triplicate and contained: 10 μL 5X Phusion HF buffer, 1 μL dNTPs (10 mM), 5 μL of sample specific, mixed forward and reverse Unitag-Barcode primer, 0.5 μL Phusion Hot Start Polymerase (Thermo Fisher Scientific, Waltham, MA, United States), 28.5 μL nuclease-free water and 5 μL DNA template. The PCR program was initial denaturation at 98°C for 30 sec, followed by five cycles of denaturation at 98°C for 10 sec, annealing at 52°C for 20 sec, elongation at 72°C for 20 sec, and a final extension step at 72°C for 7 min. PCR products (∼350 bp) were purified using the HighPrep PCR product purification kit (MAGBIO GENOMICS, Gaithersburg, MD, United States) and quantified using the Qubit fluorometer BR assay kit (Molecular Probes by Life Technologies, Thermo Fisher Scientific, Waltham, MA, United States). Equimolar amounts of purified PCR amplicons were pooled into libraries and sent for sequencing using the Illumina MiSeq platform (GATC-Biotech, Konstanz, Germany).

### Sequencing Data Analysis

Raw paired-end MiSeq sequencing reads were analyzed using the NG-Tax pipeline (Ramiro-Garcia et al., 2018) by filtering to reads with perfectly matching primers and barcodes, which were used to demultiplex reads by sample. Forward and reverse reads were both truncated to 100 nt and concatenated. Unique sequences (operational taxonomic units, OTUs) occurring above a minimum 0.1% relative abundance threshold per sample were picked, and subjected to non-reference based chimera checking, where the parent sequence needed to be more abundant by a 0.5 ratio than the chimeric sequence. Taxonomy was assigned to OTUs using a customized version of the SILVA_128_SSU Ref database (Quast et al., 2013). Three samples from the Plates experiment, namely Rha_10x, All_50x and P18II did not pass quality criteria and were excluded from further analyses. Demultiplexed, raw reads have been deposited at the European Nucleotide Archive (ENA) under accession number PRJEB31820^1^.

### Prokaryotic Community Analysis

The resulting biom tables and tree files were analyzed in R version 3.4.3^2^ using the phyloseq package version 1.20.0 (McMurdie and Holmes, 2013) and the microbiome package version 1.1.2 (Lahti et al., 2017) for data import, storage, quality control, data transformations, subsetting, ordination methods and diversity analyses. OTUs classified as Chloroplasts were discarded from the analysis.

Diversity indices for total and viable community fractions were estimated as implemented in phyloseq, and significance was tested using Kruskal–Wallis rank sum test. DESeq2 (Love et al., 2014) as implemented in phyloseq was used to normalize the OTU table of viability-test samples and to detect differentially abundant taxa in duplicate samples. OTUs with p_adj_ < 0.01, corrected for multiple testing, were considered significantly differentially abundant.

The ape package version 5.0 (Paradis et al., 2004) was used for phylogenetic tree handling, and the picante package version 1.6-2 (Kembel et al., 2010) was used to calculate Faith’s phylogenetic diversity. Non-parametric tests on medians of phylogenetic diversity per sample group (Seawater, Sponges, Plates, MiniColumns and Liquid-Solid) were performed using the Mann–Whitney test (Mann and Whitney, 1947). Sequence counts were transformed to relative abundance per sample, and distances between samples were calculated using both weighted and unweighted UniFrac distances (Lozupone et al., 2011) as implemented in phyloseq. Principal Coordinates analyses on weighted UniFrac distance metrics were performed to visualize beta diversity differences for sample groups. The degree of dispersion in beta diversity was calculated using the betadisper function, and the adonis test with 999 permutations was used to test significance of associated variables affecting the clustering as implemented in the vegan package version 2.4-5 (Oksanen et al., 2016). Venn-diagrams displaying shared and unique OTUs per sample group were created using Venny 2.1 (Oliveros, 2007) and redrawn using Microsoft PowerPoint. Relative abundances of the top 100 cultivated taxa were visualized using the pheatmap package version 1.0.8 (Kolde, 2012) and refined using Adobe Photoshop.

Canonical correspondence analysis (CCA) as implemented in phyloseq was conducted on relative abundance data to analyze the effect of the variable Medium dilution on the Plates sample set and of the variable location within the MiniColumns sample set. Significance values were calculated using the anova.cca function as implemented in vegan. To assess the overall influence of aeroplysinin-1, relative abundances of taxa were calculated for the sum of reads of samples with or without the antibiotic. The linear model relating oxygen concentration and phylogenetic diversity was fitted as implemented in the R stats package. Ggplot2 version 2.2.1 (Wickham, 2016) was used for data visualization. Full code and input files are available on GitHub^3^.

## Results

In total, 4 196 239 high-quality, denoised 16S rRNA gene sequences were obtained, with a minimum of 2 390 and a maximum of 344 380 sequences per sample. These sequences were clustered into a total of 587 operational taxonomic units (OTUs).

### Viability of Microorganisms After Cryopreservation

**FIGURE 2:**
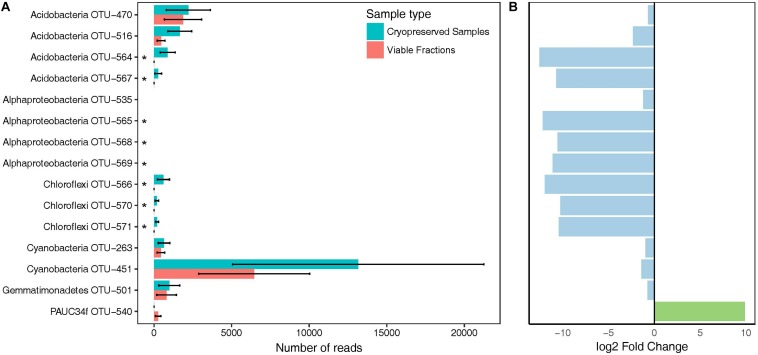
Bacterial taxa showing differences in abundance in viable fractions (PMA Treatment) as detected by DESeq2. **(A)** Total read counts of significantly different (p_adj_ < 0.01) taxa from duplicate cryopreserved (turquoise) and viable (red) fractions (PMA Treatment). Asterisks mark taxa falling below the detection threshold of 0.1% relative abundance in the viable fractions. **(B)** Log2 fold change of OTUs that increased (green) or decreased (blue) in viable fractions compared to cryopreserved samples. Bars correspond to the OTU names in **A)**.

The viable fraction of cryopreserved *A. aerophoba* cell suspensions was analyzed by comparing the differences in community composition of the total (cryopreserved samples) and viable fractions (PMA treatment of cryopreserved samples). The number of OTUs observed in the viable fractions was not significantly different to that of the total communities (*n* = 104 vs. 109; *p* = 0.1213, Kruskal–Wallis rank sum test). Similarly, no statistically significant difference was observed for the phylogenetic diversity (PD_avg_ = 11.5 *vs*. 11.9; *p* = 0.1213). A total of 100 OTUs (88.5%) were shared between viable and total communities, whereas nine and four OTUs were uniquely detected in cryopreserved samples and viable fractions, respectively. We used DESeq2 to identify 15 OTUs that were significantly (p_adj_ < 0.01) differentially abundant in viable fractions (Figure 2). In total, these OTUs accounted for a cumulative relative abundance of 12.4% in the cryopreserved community, and 4.5% in the viable fraction. Furthermore, of these 15 OTUs, only Cyanobacteria OTU-451 and several acidobacterial OTUs represented predominant members of the sponges’ cryopreserved prokaryotic community that showed a notable decrease in relative abundance in the viable fractions after PMA treatment. PAUC34f OTU-540 was the only taxon that showed a significant increase in relative abundance in the viable fractions, however, this OTU was absent or below the detection threshold in the total cryopreserved communities.

### Cultivation Overview

*Aplysina aerophoba*-derived bacteria were grown in three different cultivation experiments resulting in 63 samples referred to as “cultivation samples”: (a) a variety of plates with gelrite-solidified media tailored toward the cultivation of Poribacteria, (b) a “Liquid-Solid”cultivation where bacteria were incubated in liquid medium and subsequently transferred to petri dishes containing the same medium solified with gelrite and covered with the same liquid growth medium, and (c) in “MiniColumns” (Figure 1). Table 2 provides an overview of the 39 “Plates,” four “Liquid-Solid” and 20 “MiniColumns” samples derived from the cultivation experiments.

**FIGURE 3:**
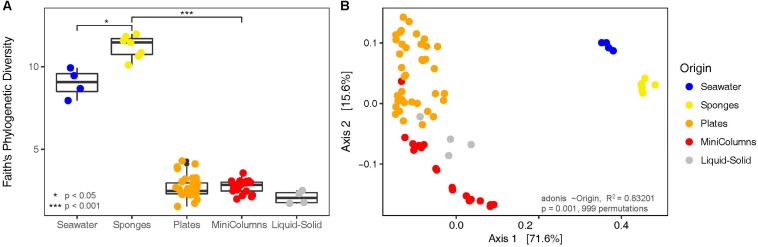
Diversity analyses of the bacterial communities derived from environmental samples and the different cultivation approaches. **(A)** Faith’s Phylogenetic Diversity and **(B)** Principal Coordinates Analysis on weighted UniFrac distances.

**FIGURE 4:**
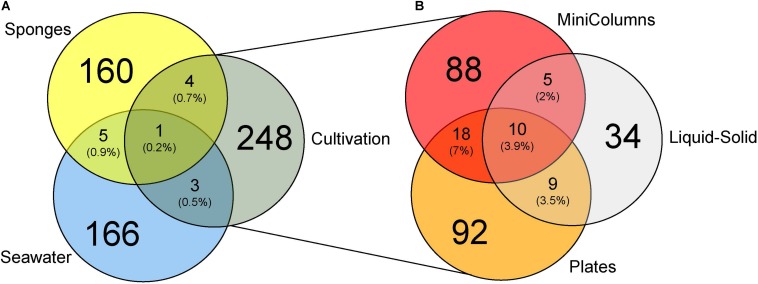
Venn diagrams displaying **(A)** OTUs unique and shared (% of number of OTUs) between environmental samples and cultivation experiments and **(B)** OTUs unique and shared between the three different cultivation experiments.

In order to compare the phylogenetic richness of prokaryotes within *A. aerophoba*, the surrounding seawater and the communities that were enriched within the different cultivation approaches, Faith’s phylogenetic diversity was calculated (Figure 3A). Phylogenetic diversity was substantially lower in samples derived from any of the cultivation experiments, indicating a strong bias toward cultivating only certain phylogenetic groups. Interestingly, this was contrasted by the observation that the total number of unique OTUs recovered by all cultivation experiments together was higher compared to that observed for the original sponge samples (Mann–Whitney Test *p* < 0.05) (Figure 4A). It should be noted, however, that the number of samples derived from cultivation experiments was much higher (*n* = 63) as compared to the number of sponge samples analyzed (*n* = 7).

Sponges, seawater and cultivation samples showed little overlap in prokaryotic community composition (Figure 3B), and sample type significantly (adonis *R*^2^ = 0.83201, *p* = 0.001) explained the separation. We observed this large heterogeneity with respect to prokaryotic community composition for cultivation samples (Figure 3B). Prokaryotic communities derived from plates were found well separated from the MiniColumn samples, whereas the Liquid-Solid samples were positioned in between. Even though large heterogeneity of recovered bacteria was also observed within each of the cultivation method sample groups (Plates, Liquid-Solid, MiniColumns), the degree of dispersion in beta diversity was not significantly different (permutation test of beta dispersion F.Model = 2.1915, Pr(>F) = 0.084). This means that all groups exhibited similar levels of variation within the group, confirming that the differences between cultivation method sample groups were caused by the cultivation method and not by chance. For unweighted Unifrac analysis, a similar result was obtained (Supplementary Figure S4).

In total, the three cultivation experiments yielded 256 cultured OTUs from seven bacterial phyla, namely Actinobacteria (1.2% of cultivated OTUs), Bacteroidetes (5.1%), Firmicutes (29.7%), Planctomycetes (3.1%), Proteobacteria (56.6%), Tenericutes (0.4%) Verrucomicrobia (0.4%) and an unidentified phylum related to the Planctomycetes (3.5%). Amongst the Proteobacteria, Alphaproteobacteria (44.5%) and Gammaproteobacteria (10.9%) dominated the cultivated fraction. No Poribacteria OTUs were detected in any of the cultivation experiments. On average, 17 OTUs were detected per cultivation sample, with a minimum of seven and a maximum of 36 OTUs. This confirmed that the number of OTUs recovered per colony PCR pool from the plates was in the range of the expected numbers based on the fact that on average PCR products of 40 colonies were pooled into one sample.

Of the 60 most abundant OTUs associated to *A. aerophoba*, only one *Synechococcus* cyanobacterial OTU was also detected in seawater, and none in the cultivated fractions. Among the 60 most prominent seawater OTUs, three *Synechococcus* cyanobacterial OTUs were also detected in sponges, and two [*Ruegeria* sp. (Alphaproteobacteria) and *Idiomarina* sp. (Gammaproteobacteria)] were recovered by cultivation. Of the 256 OTUs recovered by cultivation, four OTUs, classified as *Pseudovibrio* sp. (Alphaproteobacteria), *Halomonas* sp. (Gammaproteobacteria), Flavobacteriaceae family and *Lutimonas* sp. (both Bacteroidetes) were shared between the sponge tissue and the cultivated fraction (Figure 4A). Another three OTUs, *Halomonas* sp*., Idiomarina* sp. and *Hyphomonas* sp. (Alphaproteobacteria) were shared between the seawater and the cultivated fraction. One OTU, *Ruegeria* sp. (OTU-3), the overall most dominant OTU within the Plates approach, was shared between seawater, sponge tissue and the cultivated fraction.

**FIGURE 5:**
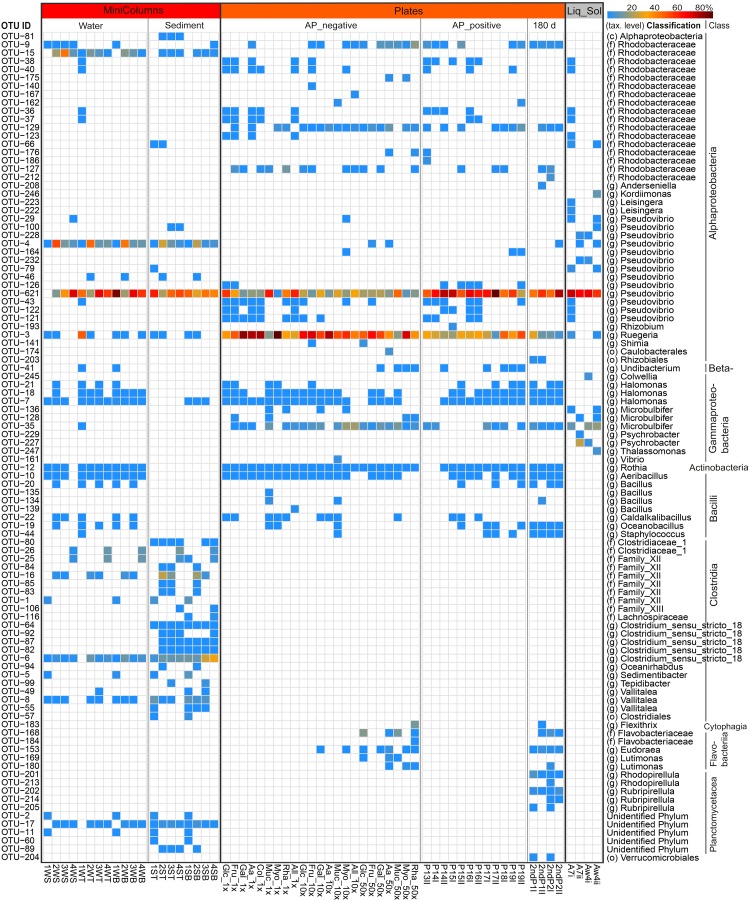
Heatmap showing the relative abundance of the top 100 cultivated OTUs, representing 94–100% of the total number of reads in individual samples. The color scale shows relative abundance (%) within each sample. Rows of the heatmap are ordered by taxonomic classification, and OTUs were classified up to class (c), family (f) or genus (g) level. SampleIDs reflect cultivation media and can be obtained from Table 1.

The different cultivation approaches shared merely 10 OTUs, classified as *Ruegeria* sp*., Pseudovibrio* sp*., Microbulbifer* sp. and five other Rhodobacteraceae family (Alphaproteobacteria) OTUs, indicating that most bacteria were only obtained by one cultivation approach (Figure 4B). The MiniColumns and the Liquid-Solid approach shared another five OTUs from the Rhodobacteraceae family, four of which were further classified as *Pseudovibrio*. The Liquid-Solid and Plates approaches shared further nine OTUs, five of which belonging to the Rhodobacteraceae family (three *Pseudovibrio*), and another four to the gammaproteobacterial *Microbulbifer* genus. The MiniColumns and the Plates approach shared another 18 OTUs belonging to the three phyla Firmicutes, Proteobacteria (Alpha, Beta and Gamma) and Actinobacteria. Interestingly, all of these except for one *Pseudovibrio* OTU were detected only in the water samples of the MiniColumns, as well as on the plates.*Pseudovibrio* sp. OTU-621 was recovered from all but one cultivation derived samples and dominated the cultivable fractions from 3.0 to 89.7% relative abundance per sample (Figure 5). This OTU was also detected in sponge samples with an average of 0.3% relative abundance. In addition to the high relative abundance of OTU-621, a large number of other OTUs from the genus *Pseudovibrio* were recovered from a variety of growth media and conditions. Further, *Ruegeria*, Bacilli (Firmicutes) and various *Halomonas* OTUs were also found in samples derived from many conditions. However, many recovered taxa had a preference for one of the cultivation methods. This difference was especially pronounced with respect to the exclusive presence of Clostridia (Firmicutes) OTUs in the anaerobic sediment fractions of the MiniColumns. All sediment fraction samples (especially of MiniColumn2) were also enriched for OTUs that could not be assigned to a phylum using the SILVA_128_SSU Ref database (OTU-2, 11, 17, 60, 89). The most abundant of these OTUs (OTU-17, up to 5.8% relative abundance in sediment fractions of MiniColumn2) exhibited maximally 97.5% nucleotide sequence similarities to unidentified Planctomycetes, recently detected by deep-cultivation from the calcareous sponge *Clathrina clathrus* (e.g., GenBank accession no. CP036425.1). Furthermore, an OTU belonging to the rarely cultivated phylum Tenericutes was detected with approximately 2% relative abundance in three samples derived from MiniColumn3. On the other hand, Flavobacteriia (Bacteroidetes) OTUs and *Microbulbifer* OTUs were nearly absent in the MiniColumns, but were recovered at high relative abundances from plates. A prolonged incubation time of 180 days led to the recovery of members of the Verrucomicrobia and Planctomycetes on plates. The Liquid-Solid approach resulted in high recovery of *Microbulbifer* and other Gammaproteobacteria from the genus *Psychrobacter*, of which the latter was detected solely in the Liquid-Solid cultivation.

### Impact of Aeroplysinin-1 (AP), Medium Dilution and Carbon Sources in the Plates Experiment

From the different plates 1758 colonies were picked, including 1129 colonies from the AP_negative set and 629 colonies from the AP_positive set. Aeroplysinin-1 presence led to a lower number of colonies and impacted their size and morphology. Most colonies were small and translucent on AP_positive plates, whereas on AP_negative plates larger and partly pigmented colonies could be observed (Supplementary Figure S1). Since the final concentration of DMSO was only 0.3% in the inoculum suspension, it is probably safe to assume that the lower yield of colonies on AP_positive plates was due to the toxicity of the antibiotic.

**FIGURE 6:**
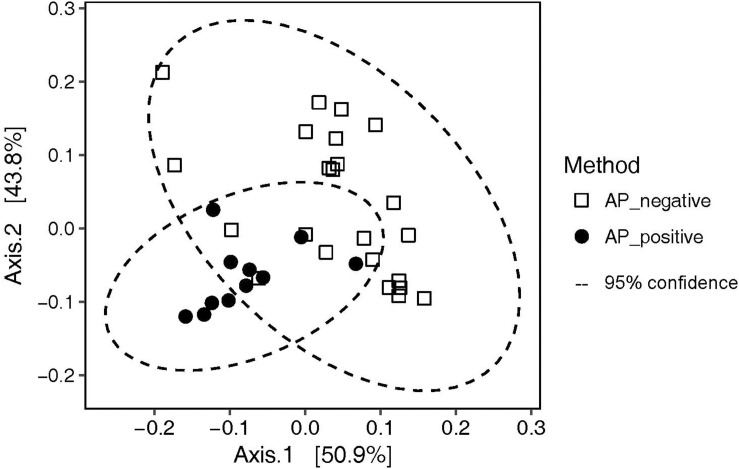
PCoA analysis on weighted UniFrac distances illustrating the effect of incubation with (AP_positive) and without (AP_negative) aeroplysinin-1 on the cultivated communities recovered from the plates.

The 16S rRNA gene of 1463 colonies could be successfully PCR-amplified and sequenced. While Bacteroidetes were completely inhibited by AP, most other taxa grew also in the presence of the antibiotic (Figure 5). The *Rhizobium* (Alphaproteobacteria) OTU-193 on P15I was the only OTU exclusively detected on an AP_positive plate. Overall, the addition of AP resulted in a consistently higher (61% ± 17% vs. 27% ± 17%) relative abundance of *Pseudovibrio* sp. OTU-621, while reducing the relative abundance of *Ruegeria* sp. OTU-3 from 54% ± 20% to 30.4% ± 13% (Supplementary Figure S2). PCoA analysis on weighted UniFrac distances revealed that AP significantly (adonis *R*^2^ = 0.25784, *p* = 0.001) affected the composition of OTUs recovered (Figure 6).

**FIGURE 7:**
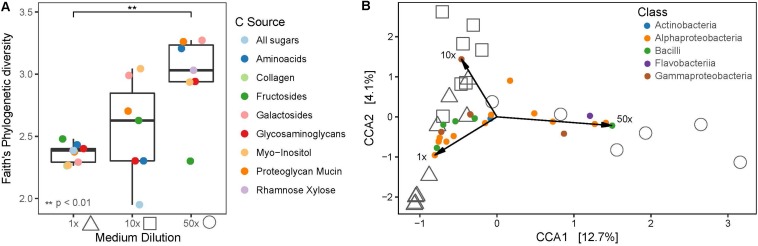
**(A)** Faith’s phylogenetic diversity of different medium dilutions and carbon sources. **(B)** Canonical correspondence analysis (CCA) biplot of cultivated bacterial communities on plates and indicative OTUs, colored at Class level. Shapes indicate medium dilutions (triangle: 1x, square: 10x, circle: 50x).

To assess the influence of medium dilution and carbon source, samples from the Plates experiment were subset to only AP_negative since for this part of the dataset the variables medium dilution and carbon source could be consistently assigned. In total, 1x diluted media yielded 40 OTUs from the phyla Actinobacteria, Firmicutes and Proteobacteria, where else 10x and 50x diluted media resulted in 50 and 38 OTUs, respectively, from Actinobacteria, Bacteroidetes, Firmicutes and Proteobacteria. Overall, phylogenetic diversity increased significantly (*p* < 0.01) in 50x diluted media compared to the 1x diluted media, whereas carbon source did not significantly impact the recovered bacterial diversity (Figure 7A). Consistently, medium dilution (adonis *R*^2^ = 0.25771, *p* = 0.015) and not carbon source (adonis *R*^2^ = 0.47646, *p* = 0.165) was the significant variable explaining community composition patterns in constrained coordinate analysis (Figure 7B).

Certain bacterial classes were associated with specific medium dilutions such as Flavobacteriia or Bacilli, which were mainly recovered on highly diluted or less diluted media, respectively. Proteobacteria were cultivated on all medium dilutions. Phylogenetic diversity was highest in the 50x diluted media containing galactosides, followed by mucin and amino acids, of which the latter also showed the highest relative abundance of Flavobacteriia.

### The Liquid-Solid Cultivation Approach

Since on plates, the 50x diluted amino acids medium (Aa_50x) resulted in high phylogenetic diversity diversity of cultivated bacteria and supported the growth of Flavobacteriia, this medium was selected to create liquid cultures. We observed biofilm formation on the bottom of the liquid culture wells after 7 days, upon which we transferred the cultures to solid Aa_50x medium and overlaid it with the same liquid medium for a total of 4 weeks of incubation. The cultures were dominated by *Pseudovibrio* OTUs after 7 days as well as after 4 weeks. Nonetheless, the Liquid-Solid cultivation resulted in the recovery of genera that were not detected in other cultivation methods, such as the gammaproteobacterial genera *Colwellia*, *Thalassomonas*, and *Psychrobacter*, as well as the alphaproteobacterial genera *Leisingera* and *Kordiimonas* at low relative abundances. Faith’s phylogenetic diversity was not significantly different than for the other cultivation approaches (Figure 3A), indicating a similar level of recovered species richness.

### Impact of Oxygen Concentrations in the Winogradsky Columns

**FIGURE 8:**
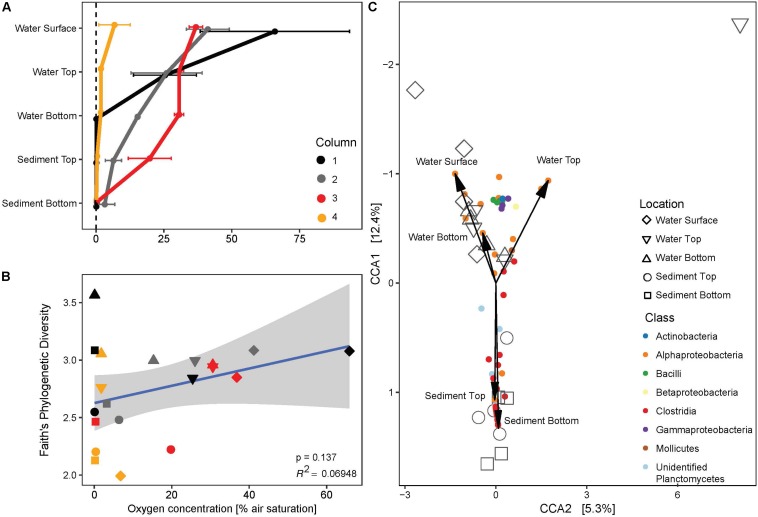
**(A)** Vertical profiles of dissolved oxygen concentration along the four replicate MiniColumns. **(B)** Regression analysis of phylogenetic diversity and oxygen concentration. Gray area marks 95% confidence region. Colors of symbols are as indicated in panel **(A)**, whereas shapes indicate location in the column as indicated in the legend to panel **(C)**. **(C)** CCA triplot of enriched bacterial communities (large symbols) in relation to bacterial OTUs that best explain the ordination (colored at Class level) and location in the MiniColumns (arrows).

At the end of the 130 days incubation period, the oxygen gradient along the depth in the MiniColumns was measured. The oxygen concentration in the headspace of all columns was 88.4% (±6.4%) air saturation, and the four replicate columns had different oxygen profiles along the column: MiniColumn4 was completely anaerobic at the top of the water column, while other columns had oxygen concentrations around 30–40% at the water surface with a gradual decrease to complete anoxia in the sediment fractions (Figure 8A). The negative control maintained a 100% oxygen saturation along the whole depth of the column (data not shown). Despite visual differences amongst the columns (Supplementary Figure S3), identical locations in different columns promoted the growth of very similar bacterial communities (Figure 5). Location within the column was the main driver of community profile differences, with a range of Clostridia OTUs and unidentified OTUs related to the Planctomycetes representing indicative taxa for the anaerobic sediment fractions of the columns (Figure 8C). Linear model regression analysis revealed a positive, though not significant correlation of oxygen concentration with phylogenetic diversity (PD). Both high (PD = 3.56) and low (PD = 1.99) phylogenetic diversities were found for anaerobic conditions, whereas microaerophilic and aerobic parts of the columns enriched for intermediately diverse communities (Figure 8B).

## Discussion

In recent years, culture-independent methods have by far outnumbered cultivation-based efforts to investigate the prokaryotic diversity in sponges. Even though multi-omics based methods can unravel numerous functional, taxonomic and ecological traits of sponge associated prokaryotic communities (Taylor et al., 2007; Thomas et al., 2016; Slaby et al., 2017), these findings remain hypothetical unless validated by experimental evidence. However, valuable insights from using molecular tools can be used to create novel cultivation conditions (Gutleben et al., 2018) in the quest to improve cultivability of sponge associated microorganisms.

### Sponge-Associated Microorganisms Presumably Remain Viable After Cryopreservation

Since sponge-associated microorganisms remain mostly recalcitrant to cultivation efforts, we aimed to investigate if the often necessary cryopreservation impacted the viability of sponge associated microorganisms. We did not detect significant differences between the total and viable communities after cryopreservation. We thus conclude that the majority of sponge-associated microorganisms survive sample processing and storage at −80°C for several months and that their current uncultivability is most likely not due to cryopreservation. Nonetheless, these results should be interpreted carefully, since the uncultivability of sponge-associated microorganisms makes it difficult to validate the viability staining techniques applied, which has been shown to be necessary in some cases (Chang et al., 2010; Emerson et al., 2017). To the best of our knowledge, only Esteves et al. (2016) analyzed the viable fraction of two Australian sponge species, after tissue separation and cell fractionation to obtain microbial pellets. Overall, they observed a loss of OTUs of up to 51% in the viable fraction, whereas we observed only a marginal loss. This difference might be due to the many steps of filtration and centrifugation involved in the cell fractionation as done by Esteves et al. (2016), which might negatively affect the viability of microorganisms, as well as lead to the loss of rare taxa.

### Most Cultivated Taxa Differ From Sponge-Associated Bacteria

Here, we described three different strategies to cultivate sponge tissue-derived bacteria, which all resulted in the recovery of communities with a very different composition compared to the original sponge samples and surrounding seawater samples (Figure 3). As negative controls in the cultivation experiments consistently showed no microbial growth, we conclude that these cultivated OTUs must be derived from rare members of the sponge community that were below the applied detection threshold.

Defining cultivability as the number of taxa that could be cultured divided by the total number of taxa detected in the sponge tissue, we report here a recovery of 2% of the sponge-associated taxa by cultivation. This falls into the range of other sponge-microbe cultivation experiments (Sipkema et al., 2011; Lavy et al., 2014; Keren et al., 2015; Esteves et al., 2016; Versluis et al., 2017), which report a recovery rate of 0.1 to 14%. Even though different sponge species harbor species-specific and highly divergent microbiota, it has been shown that their cultivable fractions are comparable (Li et al., 2007; Hardoim et al., 2014) since rare generalists are proliferating under laboratory conditions.

Despite employing newly designed media containing various unusual carbon sources, the novelty of recovered isolates was moderate. Except for the unidentified OTUs related to Planctomycetes, which were not obtained as pure isolates, the 100 most predominant cultivated OTUs had a sequence identity of >98% to the closest cultivated relative in the NCBI database. This may be partially explained by the high degree of sequence conservation of the V4 region of the 16S rRNA gene (Sun et al., 2013). Nevertheless, five OTUs that were present in the sponge tissue were also recovered by cultivation. Amongst these shared taxa, the alphaproteobacterial *Pseudovibrio* and *Ruegeria* OTUs were the most frequently cultivated. *Pseudovibrio* species represent low abundant sponge symbionts which are known as versatile, opportunistic bacteria capable to adapt to a wide range of cultivation conditions (Muscholl-Silberhorn et al., 2008; Bondarev et al., 2013; Versluis et al., 2017; Fróes et al., 2018). With this study we could furthermore add aeroplysinin-1 to the list of antibiotic resistances exhibited by *Pseudovibrio* species (Versluis et al., 2017). The genus *Ruegeria* is part of the abundant seawater-dwelling *Roseobacter* lineage (Buchan et al., 2005; Wagner-Döbler and Biebl, 2006) and has frequently been isolated from sponges and other marine environments (Mitova et al., 2004; Muscholl-Silberhorn et al., 2008; Esteves et al., 2013; Rua et al., 2014). Two other shared OTUs (OTU-168, 169) belong to the class Flavobacteriia, members of which are regularly detected in and isolated from marine sponges, however, their roles as sponge symbionts remain to be investigated (Lavy et al., 2014; Montalvo et al., 2014; Horn et al., 2016; Yoon et al., 2016; Versluis et al., 2017).

Increased incubation time of 180 days resulted in a broader detected cultivated bacterial diversity, as evidenced by the detection of Planctomycetes and Verrucomicrobia OTUs on plates. To our knowledge, this is the first report of an *A. aerophoba* derived member of the Verrucomicrobia phylum detected during cultivation, and OTU-204 exhibits only 91% sequence similarity to other cultured sponge-derived Verrucomicrobia: *Rubritalea marina* (Scheuermayer et al., 2006) and *Rubritalea spongiae* (Yoon et al., 2007). Planctomycetes have been obtained from *A. aerophoba* before (Pimentel-Elardo et al., 2003), however, the unidentified OTU-17, which was enriched to up to 5% relative abundance in sediment fractions of the MiniColumns, exhibited 97.5% sequence similarity to the closest cultivated neighbor, a member of the Planctomycetes recently obtained by deep-cultivation from the calcareous sponge *Clathrina clathrus* (GenBank: CP036425.1, Wiegand et al., 2019). Other close uncultured phylogenetic relatives of OTU-17 have been found associated to diseased tissue of a Caribbean coral (AF544881, 96% sequence identity, Pantos et al., 2003), as well as in seawater from 3000 m depth close to the Mariana Trench (AB703899, 96% sequence identity, Nunoura et al., 2015).

In this study, we extended the attempt of Lavy et al. (2014) to design cultivation media for the candidate phylum Poribacteria. The predicted genetic potential for the utilization of urea and organic phosphorous, as well as the potential to degrade a variety of carbohydrates was taken into account (Siegl et al., 2011; Kamke et al., 2013) to design nine defined media. Additionally, trace metal and micronutrient solutions were tailored toward meeting potential co-factor requirements of annotated Poribacteria enzymes (Siegl et al., 2011; Kamke et al., 2013). Furthermore, all media were diluted to account for Poribacteria potentially being oligotrophs, and incubation time was prolonged to 180 days to account for potential slow growth (Vartoukian et al., 2010; Prakash et al., 2013). Gellan gum was used as solidifying agent to avoid inhibitory effects of agar (Janssen et al., 2002; Overmann, 2010). However, none of the incubation conditions applied enabled the cultivation of Poribacteria. Further adaptations of the cultivation conditions might resolve this in the future, such as selective enrichment based on the predicted Wood-Ljungdahl pathway (Siegl et al., 2011). Also, the inclusion of siderophores (Vartoukian et al., 2016) or the provision of helper strains might aid in the cultivation of this sought-after bacterial candidate phylum (Morris et al., 2008; Davis et al., 2014; Pande and Kost, 2017).

### Effects of Micro- and Macroenvironmental Cultivation Conditions on Cultivated Taxa

We observed that the sponge-derived antibiotic aeroplysinin-1 (AP) strongly inhibited bacterial growth and led to decreased cultivated diversity on the plates. The number, size and pigmentation of colonies was negatively impacted, hinting at an overall cellular toxicity of this antibiotic (Supplementary Figure S1). AP seemed to affect taxa differently, as observed by the consistently higher relative abundance of the sponge-associated *Pseudovibrio* sp. OTU-621 in the presence of AP as compared to the seawater-derived *Ruegeria* sp. OTU-3. *Ruegeria* species seem to be negatively affected by AP, which supports the notion that this antibiotic contributes to protecting the sponge from seawater-derived bacterial infections (Lipowicz et al., 2013). Furthermore, all cultivable members of the phylum Bacteroidetes were completely inhibited by the antibiotic.

Within the AP_negative set, the highest media dilutions (50x) supported the growth of the most phylogenetically diverse bacterial community. This observation supports the notion that many marine organisms require oligotrophic conditions for successful cultivation and might be inhibited by increased substrate concentrations (Stevenson et al., 2004; Hanson et al., 2007; Pham and Kim, 2012), which was also demonstrated for sponge-derived bacteria (Hentschel et al., 2001; Muscholl-Silberhorn et al., 2008). Medium dilution, not carbon source, was the only significant factor driving the development of specific communities within the Plates experiment. This could indicate that many marine heterotrophic microorganisms are equipped with the genetic potential to degrade a wide range of carbohydrates, which was reported for e.g., Poribacteria, *Pseudovibrio* or marine Flavobacteriia (Kamke et al., 2013; Barbeyron et al., 2016; Alex and Antunes, 2018), and are more influenced by the concentration of nutrients.

Overall, the recovered prokaryotic communities clustered based on the cultivation method applied, indicating that macro-environmental conditions such as liquid (water samples in MiniColumns, Liquid-Solid cultivation) or solid medium interface had a more pronounced effect than micro-environmental conditions such as carbon source. For example, the Aa_50x medium applied in plates enriched for Flavobacteriia, while in the Liquid-Solid cultivation the same medium supported the growth of gammaproteobacterial genera *Psychrobacter*, *Thalassomonas*, and *Colwellia*, which were not detected in other cultivation experiments within this study. Liquid cultivation approaches have only rarely been applied to cultivate sponge-associated bacteria (Sipkema et al., 2011), and have been shown to result in lower species diversity as compared to solid agar plates (Schoenborn et al., 2004). However, liquid cultivation can result in the recovery of OTUs not detected in other cultivation methods (Sipkema et al., 2011), which we could also observe in this study.

### Impact of Oxygen Concentrations in the Winogradsky Column Approach

To our knowledge, this is the first report applying a Winogradsky column approach to sponge-derived samples. Winogradsky columns are enclosed, self-sustaining microbial ecosystems, where chemical gradients create niches for different microorganisms and metabolic interactions are maintained (Madigan et al., 2014; Rundell et al., 2014).

During incubation, oxygen gradients ranging from moderately aerobic in the water columns to complete anoxia in the sediment fractions developed. Community composition varied between the aerated parts of the columns, which exhibited higher relative abundances of mostly aerobic bacteria such as Actinobacteria and Bacilli, and the anaerobic parts of the columns, which were dominated by Clostridia. The unidentified OTU-17 related to Planctomycetes was enriched to up to 5% relative abundance in the anaerobic sediment fractions of the MiniColumns, hinting toward an anaerobic lifestyle of this microbe. Most fractions of the MiniColumns also exhibited high relative abundances of *Pseudovibrio*, confirming the facultative anaerobic lifestyle of this bacterial genus (Shieh et al., 2004).

The overall phylogenetic diversity of enriched microorganisms was positively correlated with oxygen concentration, even though some exceptional samples exhibited high phylogenetic diversity in anoxic conditions (Figure 8). These results indicate that anaerobic cultivation approaches can yield a comparable diversity, with distinct and potentially novel recovered taxa, as indicated by Lavy et al. (2014). In their study, six out of eight novel taxa with <94% sequence identity to the closest cultivated strains were recovered from strictly anaerobic conditions. Also for *A. aerophoba* and other sponges, periodically occurring tissue anoxia has been observed, indicating a potential niche for anaerobic microbes (Hoffmann et al., 2005, 2008) and the need to further explore anaerobic cultivation techniques for culturing sponge-associated microorganisms.

## Conclusion

Based on the results presented here we conclude that sponge-associated microbes presumably remain viable during sampling, tissue separation and cryopreservation. In this study, we showed that medium dilution rather than media diversification leads to increased diversity of recovered bacterial isolates and that the sponge-derived antibiotic aeroplysinin-1 has a strong impact on the number and morphology of bacterial colonies. Overall, only five OTUs overlapped between cultivated bacteria and the sponge tissue, accounting for 2% of the bacterial richness from *A. aerophoba*.

The previously unreported use of a Winogradsky column approach for cultivating sponge microbes could enrich for novel OTUs. This indicates the potential of such stratified cultivation systems for exploring the dynamics of sponge associated prokaryotic communities independently from the host under controlled *in vitro* conditions. Winogradsky columns supplemented with different substrates could become a promising tool to investigate whether sponge-derived microbes can form self-sustainable microbial ecosystems and study their metabolic interactions across aerobic as well as anaerobic niches. To date, the majority of sponge-associated prokaryotes remain uncultivated, calling for further novel media formulations and incubation strategies in the quest to recreate conditions that resemble the sponge ecosystem and thus increase the cultivability of sponge-associated bacteria.

## Data Availability Statement

The datasets generated for this study can be found on GitHub (https://github.com/mibwurrepo/Gutleben_et.al_Culti vation_A.aerophoba_Bacteria). Demultiplexed, raw reads have been deposited at the European Nucleotide Archive (ENA) under accession number PRJEB31820 (http://www.ebi.ac.uk/ena/data/view/PRJEB31820).

## Author Contributions

The research question was formulated by JG and DS. CL and LR carried out the experiments. JG, CL, and LR performed initial data analyses, with the assistance of SS. JG wrote the manuscript and all authors contributed to its improvement.

## Conflict of Interest

The authors declare that the research was conducted in the absence of any commercial or financial relationships that could be construed as a potential conflict of interest.

## Footnotes

1 http://www.ebi.ac.uk/ena/data/view/PRJEB31820

2 https://www.r-project.org

3 https://github.com/mibwurrepo/Gutleben_et.al_Cultivation_A.aerophoba_Bacteria
