# Supplementary material for: Cultivation of Bacteria From Aplysina aerophoba: Effects of Oxygen and Nutrient Gradients
Source: Front Microbiol. 2020 Feb 19;11:175. doi: 10.3389/fmicb.2020.00175 (PMC7042410; doi:10.3389/fmicb.2020.00175)
Supplement: Supplementary file 1 [file Image_1.pdf]

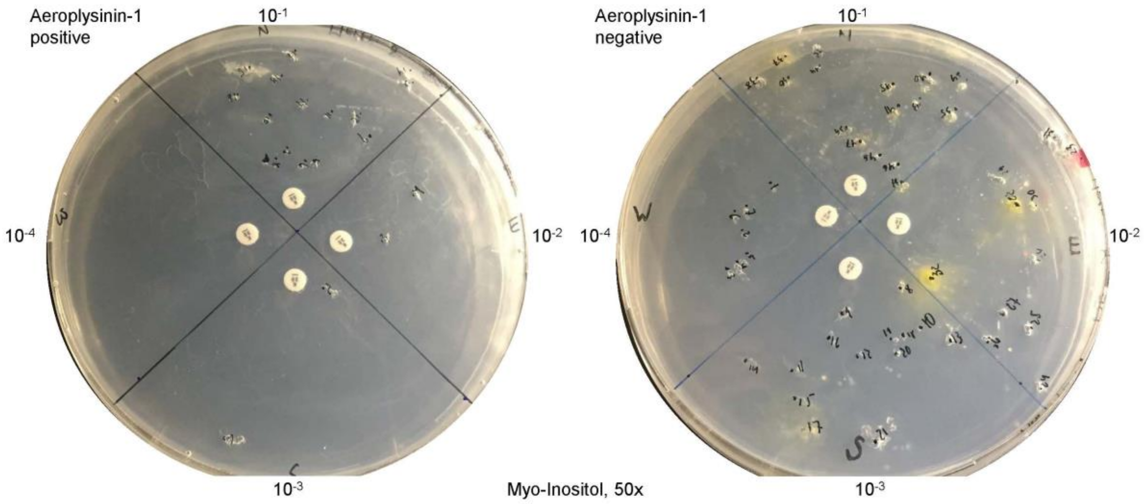

Supplementary Figure S1: Example of aeroplysinin-1 (AP) effect on colony number, size and morphology on two Plates containing the same medium.
