# Supplementary material for: Cultivation of Bacteria From Aplysina aerophoba: Effects of Oxygen and Nutrient Gradients
Source: Front Microbiol. 2020 Feb 19;11:175. doi: 10.3389/fmicb.2020.00175 (PMC7042410; doi:10.3389/fmicb.2020.00175)
Supplement: Supplementary file 2 [file Image_2.pdf]

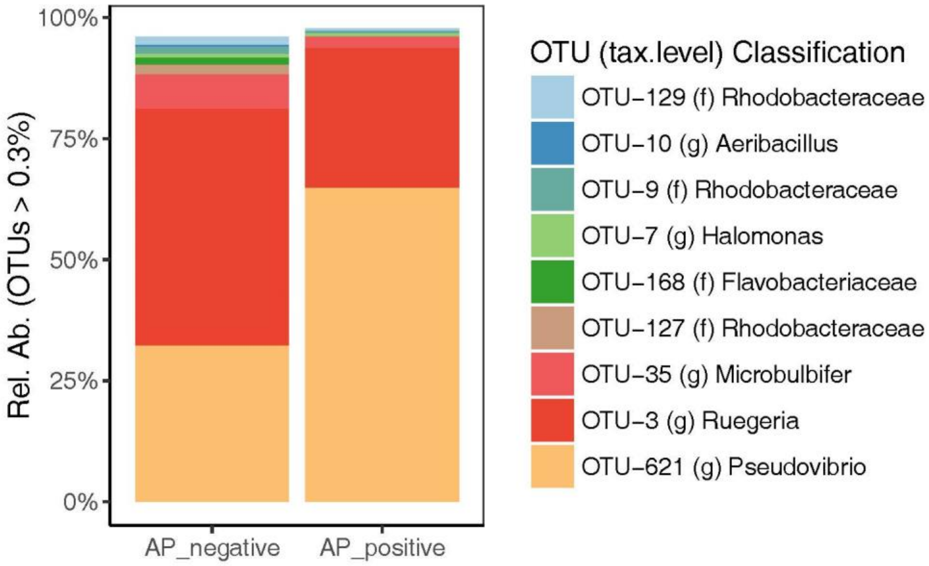

Supplementary Figure S2: Relative abundances of the most abundant taxa from the Plates, grouped from incubation without (AP_negative) and with (AP_positive) aeroplysinin-1.
