# Supplementary figures and images for: Cultivation of Bacteria From Aplysina aerophoba: Effects of Oxygen and Nutrient Gradients
Source: Front Microbiol. 2020 Feb 19;11:175. doi: 10.3389/fmicb.2020.00175 (PMC7042410; doi:10.3389/fmicb.2020.00175)

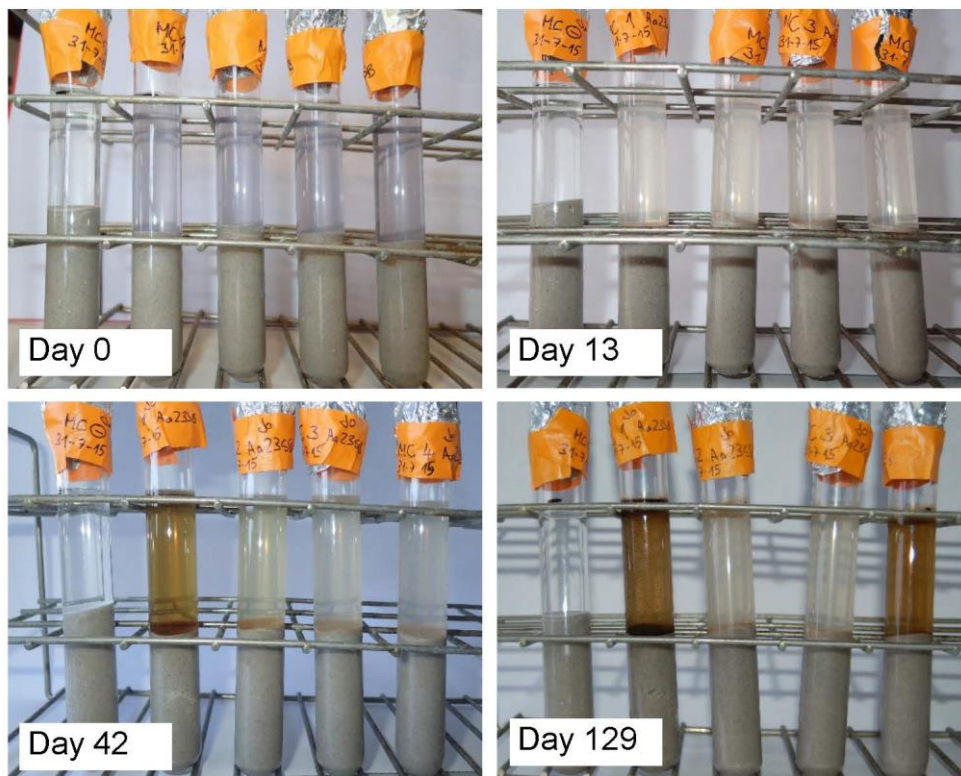

**Supplementary Figure S3:** Photographs of the MiniColumn cultivation experiment over time.

Supplement: Supplementary file 3 [file Image_3.pdf]
