# Supplementary material for: Cultivation of Bacteria From Aplysina aerophoba: Effects of Oxygen and Nutrient Gradients
Source: Front Microbiol. 2020 Feb 19;11:175. doi: 10.3389/fmicb.2020.00175 (PMC7042410; doi:10.3389/fmicb.2020.00175)
Supplement: Supplementary file 4 [file Image_4.pdf]

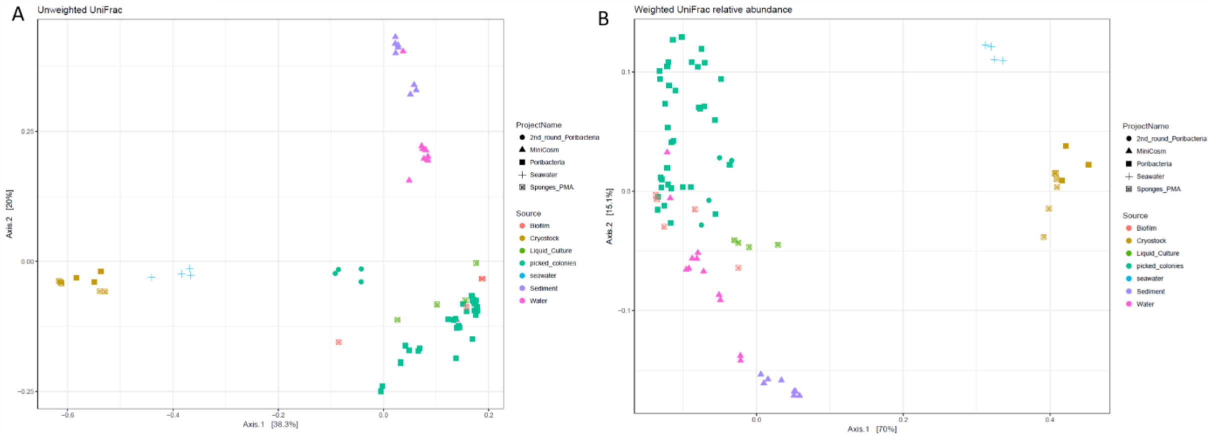

Supplementary Figure S4: PCoA analysis on unweighted (A) and weighted (B) UniFrac distances of the prokaryotic communities obtained from different cultivation experiments and the sponge inocula.
